# Surgical management of descending necrotizing mediastinitis: strategy for thoracic interference

**DOI:** 10.1186/s13019-023-02321-2

**Published:** 2023-07-12

**Authors:** Lam Xuan Nhat, Vu Huu Vinh, Chau Phu Thi, Nguyen Van Khoi

**Affiliations:** Department of Thoracic Surgery, Cho Ray Hospital, Ho Chi Minh City, Vietnam

**Keywords:** DNM, Mediastinitis, VATS, Gauze packing, Pleural cavity irrigation

## Abstract

**Background:**

The present descriptive study shares the overall experience of treating all these patients where different surgical process was adopted depending on the treatment required after carefully evaluating the risk factors and comorbidities.

**Methods:**

The present study was conducted at the Department of Thoracic Surgery, Choray Hospital, Vietnam between the period of 2010 to 2020. We have treated 95 patients altogether in this duration.

**Results:**

We were able to save most of the patients by applying thoracotomy and thoracic irrigation for most of the patients based on the observed indications that were identified immediately after the compulsory standard cervicotomy. The indication for thoracic interference was considered when the infection was deeply spread into the mediastinum and cannot get out through cervicotomy, although the most effective method of drainage was applied.

**Conclusion:**

Our statistical investigation of the patient data suggested the possible association and influence of comorbidity such as diabetes. Therefore, we recommend that in specific cases thoracotomy along with thoracic irrigation and repetitive surgical draining could be a better option to reduce the infection and the mortality rate.

**Trial registration:**

Retrospectively registered.

## Introduction

Descending Necrotizing Mediastinitis (DNM) is a serious but rare clinical condition that often occurs due to moderate to severe polymicrobial infections in the oropharyngeal region that spreads to the mediastinum region by the deep and cervical fascial plane connection. In most cases, such infections may originate from various situations that may include post-surgical conditions, cervical trauma, internal injury, odontogenic infection, regional soft tissue infection, chronic sinusitis, or any other reason [1]. Such conditions are phenotypically presented with symptoms predominantly as persistent fever, chills, sore throat, shortness of breath, cough, and chest pain radiating to the neck along with other symptoms.

Immediate diagnosis followed by treatment is inevitable due to the high mortality in such conditions [2, 3]. Considering the seriousness of the condition, different treatment algorithm has been developed to respond according to the treatment requirement [3]. Evaluation of the infections in the pleural and pericardial region is important to understand and evaluate the spread of infections [4]. Computed Tomography (CT) scan remains a reliable diagnostic tool for investigating the region of infections. Treatment measure varies depending on the spread of infections, especially in the upper and lower region of the mediastinum. Occasionally, the surgical procedure remains the only option to treat where cervicotomy or thoracotomy was opted dependent on the condition of the patient and the severity of the infection [3]. In most cases, in such a surgical process, aggressive drainage is applied to reduce infection spread and mortality [5]. In an earlier report, recommendations have been made for serial CT scan monitoring along with serial transcervical and transthoracic drainage and debridement to improve patient survival [6].

Apart from the infection severity comorbidity assessment is an important part of the therapy decision. Several comorbidities such as age, hypertension, diabetes, and other conditions should be evaluated and precautionary measures should be considered before and after the surgical procedure. Evaluation of the risk factors should be carefully done to understand the mortality risks for the patient [7]. Assessment of the risk factors that can affect the prognosis should be considered seriously during the diagnosis and treatment of DNM. Risk factors such as septic shock and associated complications should be seriously considered and evaluated [8].

Most of the reports suggested thoracotomy with severe infections that are in the mediastinum region. Often aggressive drainage is recommended to surgically treat the condition and improve the survival of the patient [9]. Along with drainage, mediastinopleural irrigation is also recommended in certain conditions [10].

The present study is aimed at demonstrating the surgical procedure outcomes for DNM in a large number of patients.

## Materials and methods

### Patients and study design

The present study was conducted at the Department of Thoracic Surgery, Choray Hospital, Vietnam and a total of 95 patients of either gender were recruited for the study. The data was assembled from the department with proper authorization and permission and the data was collected from 2010 to 2020. This is an observational and descriptive study where various parameters such as demographics parameters (age, gender), clinical and treatment (infection details, surgery-related, reason for hospitalization), associated health conditions (tonsillitis, teeth decay), and comorbidities (diabetes) were considered. The detailed clinical investigation-related factors such as pleural effusion, pericardial effusion, type of bacterial infections, and type of surgery (thoracotomy or video-assisted thoracic surgery (VATS) were also included as parameters in this study. The above patient characteristics have been summarized in Table 1. We divided the patients into two groups, 47 patients (2010–2016) and 48 patients (2016–2020) and their mortality rates were 8.51% (4/47) and 10.42% (5/48).

**Table 1:** Summary of characteristics of all 95 patients considered in the study

Patient characteristics	Type	No of patients	% of patients
Admission year	2010–2016	47	49.47
	2016–2020	48	50.53
Age	16–82	95	100.00
Gender	Male	71	74.73
	Female	24	2.26
Admission reason	Dyspnea	2	2.10
	False teeth falling	1	1.05
	Stuck fishbone	2	2.10
	Neck pain and selling	90	94.73
Associated health conditions	Teeth decay	14	14.73
	Tonsilities	7	7.36
	Tracheal injury	1	1.05
	Foreign body in esophagus	2	2.10
Prevailing symptoms	Fever	30	31.57
	Pleural effusion, right	11	11.57
	Pleural effusion, left	7	7.36
	Pleural effusion, bilateral	10	10.52
	pericardial effusion	4	4.2
Infection	Streptococcus	17	17.89
	*Klebsiella Pneumoniae*	8	8.4
Treatment	Thoracotomy	47	49.47
	Thoracic interference with neck incision	25	26.32
	Only neck incision	3	3.16

### Eligibility criteria

The study design considered strict eligibility criteria for study participation. The following inclusion and exclusion criteria were considered to be eligible to participate in the present study.

In this study, the inclusion criteria included the presence of the cervical abscess spreading to the mediastinum over the level of the innominate vein along with a severe infected condition, either identified clinically through a blood test or the presence of moderate to severity unilateral or bilateral pleural effusion or pericardial effusion. Those patients were excluded from the study who had confined cervical abscesses in the cervical region, above the innominate vein.

### Treatment and surgical procedure

DNM was detected following the nature of the origin, it was detected in the neck or oral cavity, and cervical interference was provided the most consideration. The descending spread of the infection into the mediastinum was carefully evaluated and the associated thoracic intervention was determined accordingly. The criteria for mediastinal involvement were considered based on the fluid or air/fluid level in the widened mediastinal below the level of the innominate vein, unilateral/bilateral pleural effusion, and/ or pericardial effusion. We applied two stages of management strategies for the patients: from 2010 to mid-2016 (June 2016) and from July 2016 to the end of the study. From 2010- to mid-2016, we aggressively performed thoracotomy as a part of the combined operation followed by cervicotomy in case of the infection was defined as mediastinal either in higher or lower compartments. We were having 46 cases in this period. All the cases were operated on by open thoracotomy.

For the patients who took the admission for treatment from mid-2016 (July 2016) to 2020, we followed a different strategy. We considered intensive wide opening and draining for cervicotomy operation followed by gauze packing. The gauze packing was replaced 3 times/day with irrigation and suction. This helped to drain well the infectious material from the upper mediastinum via the thoracic inlet. We decided on thoracic intervention for cases with infectious material in the lower mediastinal region that could not be drained through the cervicotomy incision. We had 49 cases in these particular conditions, and among them, 17 cases were not considered for intervention in the thorax, and 32 cases were considered for thoracic intervention, either by thoracotomy or VATS. For thoracic intervention cases, we just open the mediastinal pleura above and below the azygos vein and applied mediastinopleural irrigation and drainage. For cases with pericardial effusion, if thoracic intervention was required, we opened the pericardial cavity into the pleural cavity. In cases where thoracic intervention was not required, we performed subxiphoid pericardial drainage. The mediastinopleural irrigation continued till there was proper drain fluid seen. The concentration and volume of gentamycin solution used for pleural irrigation were 2 ampoules (80 mg x2) in a 500 ml saline bottle and 2000 ml (4 bottles)/24 h, respectively. For patients whose infection was managed by systemic antibiotics, no thoracic/mediastinal irrigation was required. It almost took around 7–20 days depending on the severity and the effectiveness of the irrigation and drainage process. Systemic antibiotics were administered through the intravenous route for all patients, irrespective of thoracic irrigation. It was administered for 10–14 days. So, mostly cefalosporine for 3 generation (1gr/24 h IV for 10–14 days) was used differently in cases. We obtained culture for infection in the thorax but never used antibiotic based on it, except in some of very severe cases. Fortunately, they are extremely rare and usually were attributed to other factors (very old age, late to the hospital, severe renal, cardiovascular chronic diseases, diabetes) than infection.

### Statistical analysis

All numerical data are presented as mean ± standard deviation and categorical data are presented as percentages in the present study. All statistical analyses were conducted using the Statistical Package for the Social Sciences (SPSS® version 25) and R (version 4.0) statistical software. All descriptive analysis was done with mean and median value presentation for the numerical parameters and percentages for the categorical parameters. Correlation analysis was done using Pearson’s correlation method. Vovk-Sellke Maximum p –Ratio was calculated depending on the p-value. The maximum possible odds in favor of the hypothesis H_1_ over H_0_ were considered as equals 1/ (-e p log (p)) for p ≤ 0.37 [11]. The paired sample T-tests were conducted using both the Student’s t-test and Wilcoxon t-test. The effect size for the Student’s t-test was calculated by Cohen’s d and the effect size for the Wilcoxon test was calculated by the matched rank biserial correlation. The chi-square test (Χ^2^) was done wherever necessary for the categorical parameters present in the study data.

## Results

### Patient demography

The present study included 95 patients who were admitted for treatment in the hospital between 2010 and 2020. In the present study, patients belonging to both genders were considered and were within the age between 16 years and 82 years (Table 1). The mean and median age of the patients was 52 years and 56 years, respectively (Table 1). The gender distribution of the patient population suggested that 71(74.73%) (n = 95) patients were males and 24 (25.26%) were females (Table 1).

The admission reasons noted during the hospitalizations were dyspnea (~ 2%, n = 95), false teeth falling (~ 1%, n = 95), stuck fishbone (~ 3%, n = 95), and the majority of the patients complained about neck pain and swelling (95.69%, n = 95) (Table 1). Hence, maybe neck pain and swelling could be a dominant symptom of DNM that could be associated with chest pain radiation apart from the common symptoms of infection on the upper respiratory tract, fever, chills, shortness of breath, and problem in the throat such as the sore throat.

### Associated health conditions

Each patient underwent an individual health inspection to investigate the comorbidities and to evaluate the associated health conditions. A dental health check suggested that 14 (14.73%) patients were having tooth decay. Altogether 7 (73.6%) patients had tonsillitis which could have developed due to the infection present in the patient’s throat and lungs. Only one patient (1.05%) had a tracheal injury and 2 (2.10%) patients had a foreign body in their esophagus. The presence of diabetes was observed in 18.94% (n = 18) of the total patients.

### Prevailing symptoms and infection evaluation

In this study population, 31.57% (n = 30) of the patients were presented with different grades of fever. Detailed investigations suggested that pleural effusion was observed in 28 patients which were further categorized in the right side (11.57%, n = 11), left side (7.36%, n = 7), and bilaterally (10.52%, n = 10). An abundance of right-side pleural effusion followed by bilateral pleural effusion was observed. The clinical investigation related to the pericardial effusion suggested that only 4 patients (4.2%) were having pericardial effusion.

Detailed analysis of the infection revealed that the patients were predominantly infected with various strains of Streptococcus (17.89%, n = 17), followed by *Klebsiella pneumoniae* (8.4%, n = 8).

### Treatment

**Fig. 1:**
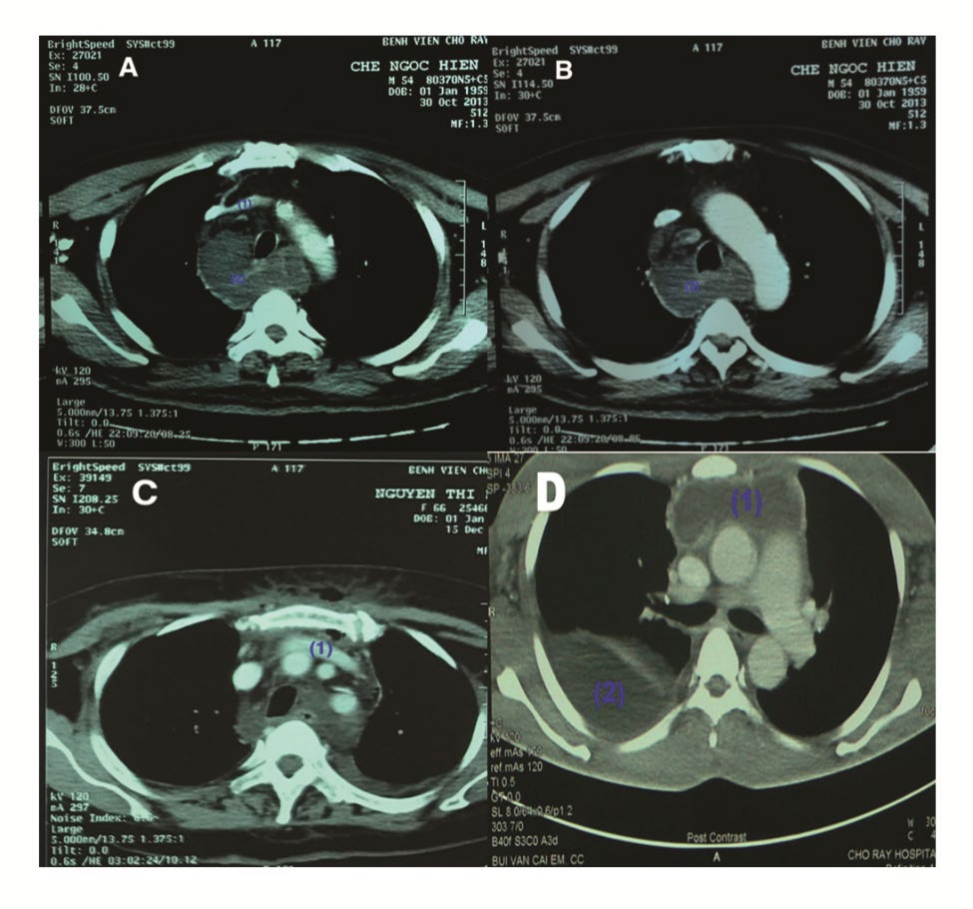
Obtained example computed tomography (CT) Scan results for the patients. (**A**) The infectious fluid (2) surrounding the trachea spread below the innominate vein (1). (**B**) The infection (2) is in the upper mediastinum. (**C**) The infection spread just to the level of the innominate vein (1). (**D**) the infection (1) spread to the lower mediastinum (carina level) plus right pleural effusion (2)

Infection was observed in most of the patients who were admitted for treatment. CT scan was done for the lungs of these patients (Fig. 1). The CT scan results show the extensive spread of infection to the thorax of the patient (Fig. 1).

We considered the innominate vein as a landmark in these surgeries. Infectious materials that spread in a descending manner but just to the level of this region were not considered as mediastinal (Fig. 1C). In case of the infections were spread clearly below this region, that was defined as mediastinal, either in the region of the upper mediastinum (Fig. 1A and B) or moving further to the lower mediastinum (Fig. 1D).

The obtained results suggest the possibility of the propagation of the infection to the entire thorax region along with the infection of the lungs or it could be confined in the mediastinum surrounding region of the trachea, i.e., below the innominate vein/artery and may deeply spread to the inferior pulmonary vein level. Figure 1 also presents the gauze packing and the cervical wound due to the surgical procedure.

**Fig. 2:**
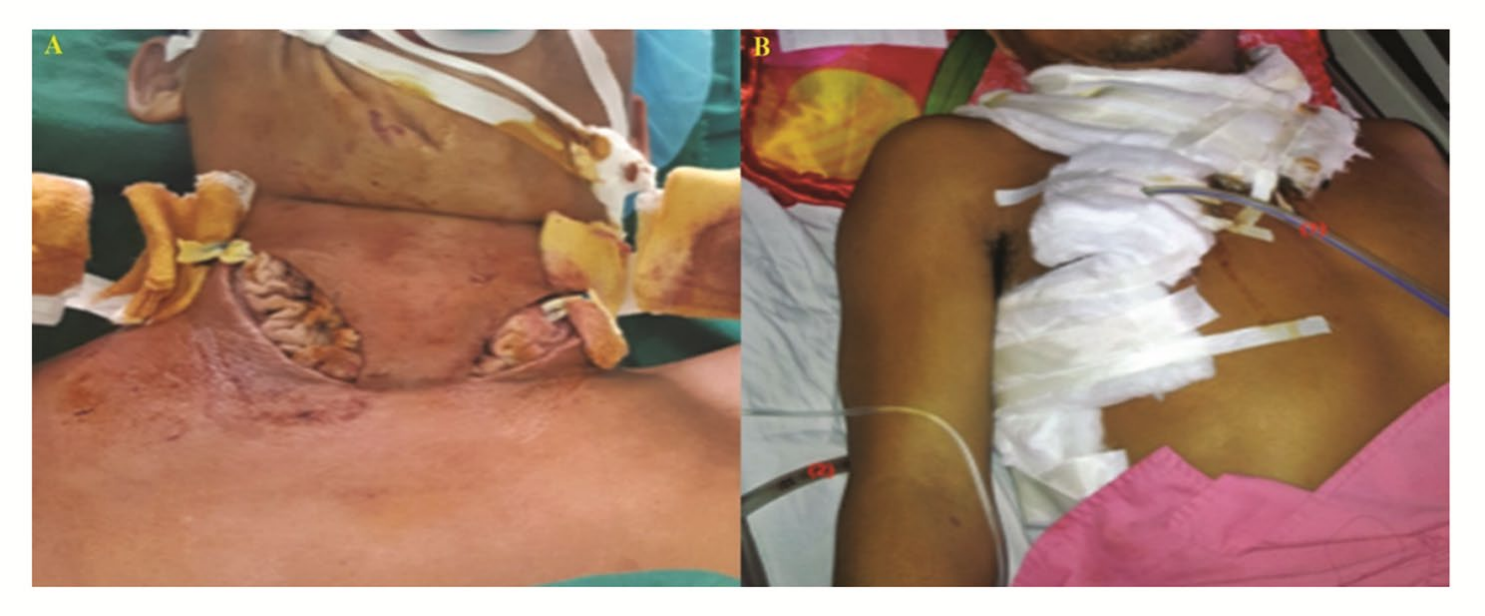
(**A**) Gauze packing in the cervicotomy incision. (**B**) Irrigating (infusion) catheter (1) and drainage chest tube (2) after thoracic interference (thoracotomy or VATS).

Due to the treatment requirement, all the patients have undergone a cervical incision. The surgical option was opted for after receiving the patients’ consent. Figure 2 presents the surgery location of a patient and the recovery. Minimally invasive VATS was conducted on 4 patients (4.2%, n = 95), and thoracotomy was conducted on 74 patients (77.89%, n = 95). Depending on the patient’s condition, immediate surgical intervention was planned for 69 patients (72.63%, n = 95).

Pericardial drainage was required for only one patient. Altogether, 30 patients (32.57%, n = 95) required mediastinal irrigation to prevent perioperative infections. Such procedures are occasionally employed to prevent serious thoracic or cardiac surgeries including open-heart surgery [12]. In most of the surgeries, Gentamicin (n = 29) was used as the irrigation solution. The outcomes of the surgery suggested a success rate of 91.57% (n = 87).

### Correlation and association analysis

**Fig. 3:**
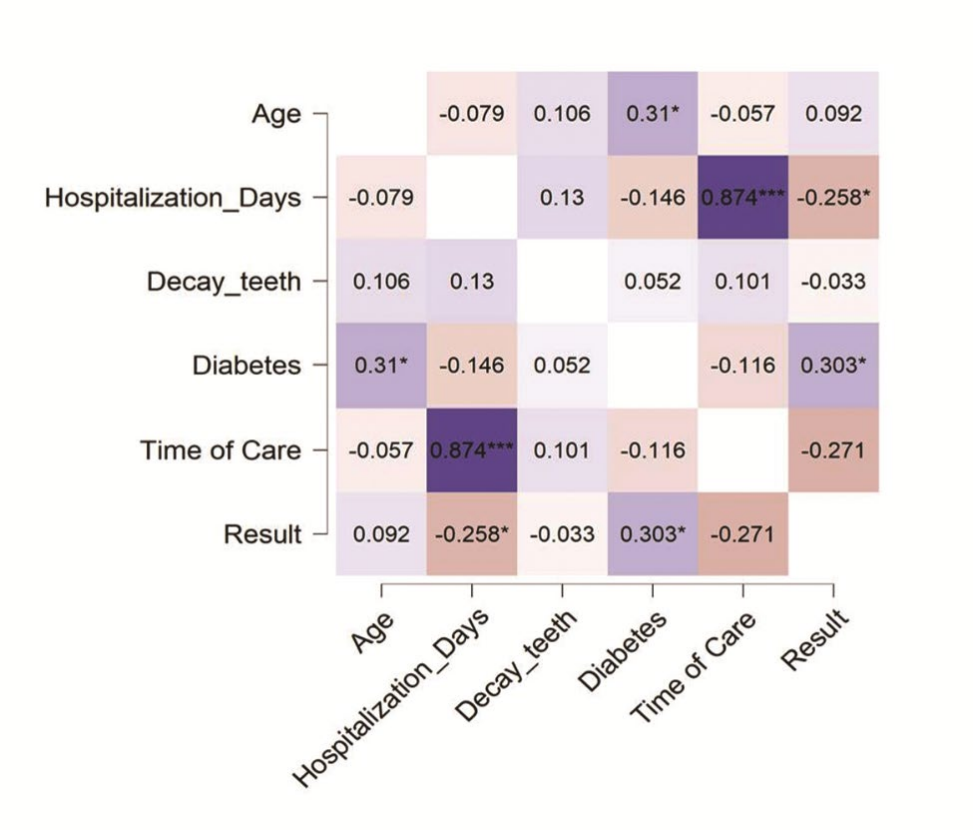
Correlation analysis of the important parameters. Heatmap presentation of correlation analysis results for age, hospitalization days, teeth decay, diabetes, results of the procedure, and time of care

The diversity of the patient’s age, the difference in hospitalization time, time of care, associated conditions, and comorbidities such as teeth decay and diabetes inspired us to investigate the possible correlation of these parameters with the surgical outcome, i.e., result and among these parameters. Correlation analysis was done for age, the number of days the patients spent for hospitalization, the presence of teeth decay, the presence of diabetes, the outcome or results of the surgical procedure, and the time of care. The outcome of the correlation analyses is presented in Fig. 3 as a heatmap. The significant observations are presented with * in the heatmap. Hence, the analysis results suggest that the number of hospitalization days and the time of care are significantly associated (Pearson’s r = 0.874***, p < 0.001). Importantly, we have observed the correlation between the presence of diabetes and possible surgical outcomes (Pearson’s r = 0.303*, p < 0.05).

**Fig. 4:**
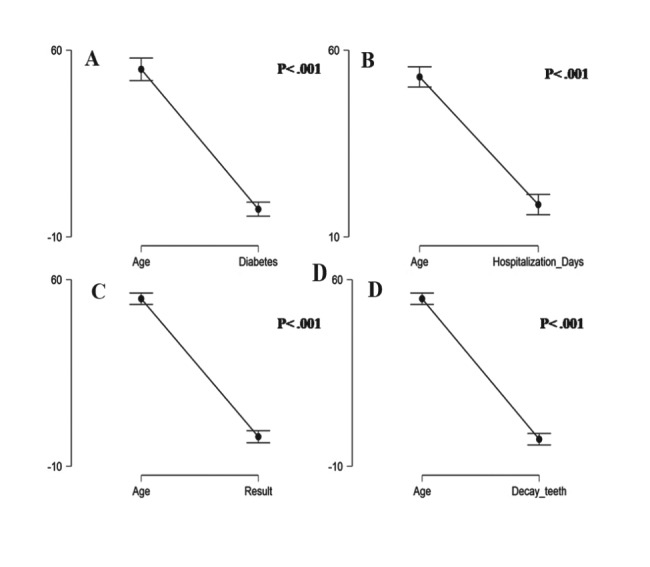
Pairwise T-test analysis of **A** age and diabetes, **B** age and hospitalization days, **C** age and surgical outcome or results, and **D** age and decay in teeth

Considering the diversity of the patients’ age in the study population, an additional analysis of the possible association of age with other parameters was conducted. A significant association was observed for age with diabetes (Fig. 4A) (p < 0.001), age with a period of hospitalization (Fig. 4B) (p < 0.001), age with surgical outcomes (Fig. 4C), and age with possible decay of teeth for the considered patient population (Fig. 4D).

Further, the study population data were segregated into two different groups where the first group contained the data collected from the study initiation till June 2016, and the second group contained the data after July 2016 till the end of the study. Such data segregation was done because the upper mediastinitis could be effectively drained via the cervicotomy incision through the thoracic inlet. So, from this stage, we intensively used packing, irrigation, and suction at the cervical incision postoperatively to avoid thoracic intervention in upper mediastinitis.

Possible associations were evaluated for patients’ age for these two groups and Chi-Square (Χ^2^) test was conducted to understand the association of the parameters. No statistical significance was observed for the patients’ age difference in these two groups. A similar analysis was extended for the patients’ gender (p = 0.4718636), and the thoracotomy procedure was considered for the patients. No statistically significant outcome was observed in any case.

## Discussion

DNM is a serious medical condition that often occurs due to severe infection and is occasionally ignored at the initial stage by the patient. Higher mortality rate all over the world has been recorded for this condition and diagnosis delay and suboptimum drainage during and after the procedure have been considered for increased mortality rate [13, 14]. The majority of the early diagnosis of this condition is done through a CT scan to understand the extent of the infection and decide on surgical strategies. An earlier report suggests proper surgical strategy with drainage and wound management can reverse the mortality rate for DNM [13]. In an earlier report, even with a normal chest roentgenogram, CT scan was recommended for early diagnosis of mediastinitis due to deep cervical infection [14]. Moreover, the application of surgical drainage and posterolateral thoracotomy-based drainage of the mediastinum with proper debridement of the neck was suggested by Mihos et al. [14]. Endo et al. provided a precise surgical guideline and management of the drainage with the categorization of the DNM [15]. A 14-year-long study on 17 patients suggested that post-oropharyngeal infection treatment and immediate detection of DNM through CT scan is important [16]. Further, DNM progress should be monitored using a CT scan and cervicotomy, and thoracotomy with proper surgical drainage may improve the treatment outcomes by up to 20% [16].

Investigation into the origin of the DNM implies that the condition originates due to the infections of multiple microbial pathogens in the pharyngeal foci. Further, the disease manifestation occurs due to the impairment of the fresh oxygen supply in the tissues and reduced immune response in the specific infected local region [17]. However, for upper tract infection, transcervical drainage may be adequate but for deeper infection, to the mediastinum and farther region, additional efforts should be made including debridement, subxiphoid incision based draining, thoracotomy as per the required evaluation of the condition [18].

Early diagnosis, thoracotomy, and surgical drainage have been advocated by other researchers also to manage the severity of the condition and reduce the mortality rate for DNM [19, 20] Moreover, for patients with compromised immunity, an aggressive surgical approach has been recommended [21].

Apart from the early detection, thoracotomy, drainage, and management of the wound, a recent study recommended applying probable minimal invasion along with multidisciplinary treatment to enhance patient outcomes [22].

In the present study, we have observed that the duration of hospitalization, the time provided for patient care, the age of the patient, and the presence or absence of diabetes may serve as crucial factors for overall patient outcomes. However, a study with a large and diverse patient population should be done from multiple centers before confirming these obtained statistically significant contributing factors.

The surgical process considered and the relevant patient outcomes in the present cases could be considered for two different periods. The observations could be considered for the surgical process conducted and treatment measures considered from the period of 2010 to 2016 and the recent period of 2017–2020. In the earlier phase (2010–2016), almost all the cases were considered for thoracic interference along with thoracic irrigation, especially for the cases having abscesses going below the innominate vein/artery. Such combined efforts of thoracic intervention and thoracic irrigation remained successful with ~ 10% mortality in comparison to the earlier outcomes recorded and reported elsewhere.

However, in the later phase (July 2016–2020), the management of the cervical wound at the post-operation stage was altered with irrigation with gauze packing of the wound and applying gauze change with intensive irrigation and suction 2–3 times /day. It was done at the deep thoracic inlet and using the gauze packing for wound management. Thus, we were successful in ceasing the spread of infection to the mediastinum and we were successful in reducing the thoracic intervention as well. Further, our experience in this aspect suggests that severe cases with massive abscesses below the innominate vein/artery may require thoracic intervention and VATS instead of thoracotomy depending on the evaluation of other conditions of the patients. Thoracic irrigation may be applied in such cases simultaneously after the assessment of the condition and surgical outcomes. The mortality rates in the two groups were 8.51% and 10.42%. The cause of death in almost all cases was severe, stubborn infection related to delayed hospitalization leading to multi-organ failure and death.

The association of the risk factors with poor disease prognosis is another concern that should be addressed by the physician apart from the early detection and surgical measures to be considered [8]. Estimating the clinical predictors such as age, neutrophil-to-lymphocyte ratio, and C-reactive protein could be vital and helpful in decision-making regarding the surgical procedure and treatment strategy development [23]. Hence, all these associated factors should be carefully considered along with precise early diagnosis, and detection of a specific surgical requirement to save more lives and reduce the mortality rates. Furthermore, the long-term results of our current study were satisfactory. All survival cases had a complete recovery and led a normal life. There were no reports of recurrence of infection in any of the patients in the study.

In the present study, we have considered different surgical approaches at different time points, especially, from 2010 to mid-2016 (June 2016) and mid-2016 (July 2016) to 2020. Our observations suggest that the outcomes are almost similar; however, the indication for thoracic intervention differed considerably. The success rate of the surgical process was higher with a lower rate of failures in the period of 2017–2020 compared to the previous period of 2011 to 2016. This lower rate of death was attained through applying intensive irrigation along with suction, and gauze packing (3 times/day) in caring for the cervical wound postoperatively. Such effort allowed for halting the fluid ejection from the cervical wound that could have continued to spread to the mediastinum. Evaluation of the associated factors in this study suggested the possible correlation with the presence of diabetes in the patients and presented in the earlier section.

## Conclusion

The present study considered 95 patients who were treated over 10 years. Our experience and the treatment outcomes suggest that early diagnosis, timely thoracic cervical gauze packing with irrigation and suction, and thoracic irrigation may reduce the mortality rate of the patients. This can reduce the requirement of thoracic intervention in cases that have descending infection just in the upper mediastinum. Apart from the spread of infection in the mediastinum region, associated factors such as age, and comorbidities must be given proper attention for improving the treatment outcomes.

## Data Availability

Not applicable.

## Abbreviations

DNM: Descending Necrotizing Mediastinitis
CT: Computed Tomography
VATS: Video-assisted thoracic surgery
